# Sarcoidosis of the medulla oblongata causing intractable hiccoughs and numbness of extremities: A case report

**DOI:** 10.1097/MD.0000000000013667

**Published:** 2018-12-14

**Authors:** Xi-yuan Chen, Zhuo-chao Ren, Xiao-jun Huang

**Affiliations:** Department of Respiratory Medicine, Zhejiang Provincial People's Hospital (People's Hospital of Hangzhou Medical College), Hangzhou, China.

**Keywords:** cardiac sarcoidosis, complete atrioventricular block, hiccoughs, medulla oblongata, neurosarcoidosis

## Abstract

**Rationale:**

Sarcoidosis is a multisystem disorder characterized by noncaseating granulomas. The nervous system is involved in 5 to 16% of the patients. However, neurosarcoidosis in the medulla oblongata presenting as hiccough is remarkably rare.

**Patient concern:**

A 55-year-old female was admitted to our hospital suffering from intractable hiccough and progressive numbness of extremities.

**Diagnosis:**

The MR imaging revealed a circumscribed mass lesion located on the medulla oblongata. The mass was hyperintense on T2-weighted images and enhanced homogeneously with gadolinium-diethylenetriamine penta-acetic acid. The cerebrospinal fluid analysis showed a moderately elevated protein content and a significant lymphocytosis 86.5%. Electrocardiogram (ECG) showed complete atrioventricular block. Bilateral supraclavicular, hilar, and mediastinal lymphadenopathy was diagnosed in a CT scan. Transbranchial needle aspiration biopsy revealed noncaseating granuloma consisting of epithelioid cells, lymphocytes, and rare multinucleated giant cells which was consistent with sarcoidosis. The diagnosis of multisystemic sarcoidosis was made.

**Interventions and outcomes:**

The patient underwent a permanent pacemaker insertion, and was successfully treated with corticosteroids.

**Lessons:**

It is important to consider neurosarcoidosis in the differential diagnosis of intramedullary lesion, since a right recognition may lead to appropriate treatment with steroids and avoid needlessly extensive surgery.

## 1 Introduction

Sarcoidosis is a multisystemic disorder of unknown etiology. Microscopically, it is characterized by the formation of noncaseating granulomas with lymphocytic infiltrates. Clinically, sarcoidosis infiltrates into practically all organs including the lung, eyes, lymph nodes, heart, and nerves. The nervous system involved occurs in patients with multisystemic sarcoidosis, ranging in frequency from 5% to 16%. In particular, isolated neurosarcoidosis is quite rare and occurs in only 1% of patients.^[1]^ We report a patient with neurosarcoidosis who presented with numbness of extremities and intractable hiccoughs. Evaluation revealed an enhancing lesion in the medulla oblongata that resolved after steroid therapy. Neurosarcoidosis in the medulla oblongata is remarkably rare and has only been reported in 4 cases.^[1–4]^

## 2 Case presentation

A 55-year-old female had been suffering from numbness of extremities for a month, and she was admitted to a hospital in our city. On admission, magnetic resonance (MR) imaging revealed a intramedullary mass lesion located on the medulla oblongata. Because of high risk, the patient refuesd medullary lesion biopsy or surgical removal and was discharged. Two months later, she was admitted to our hospital presenting with intractable hiccough and progressive numbness of extremities.

**Figure 1:**
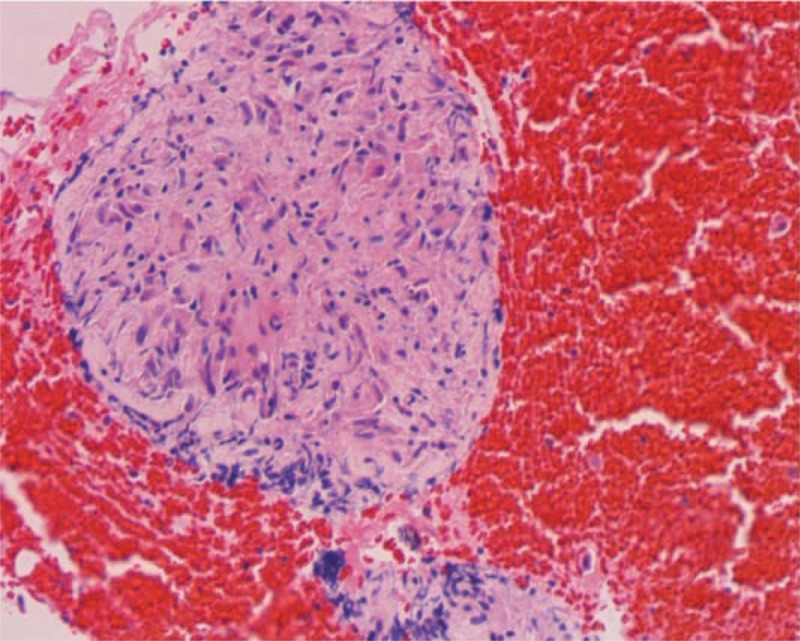
Pathological examination showing non-caseating granuloma surrounded by epithelioid cell lymphocytes. Note multinucleated giant cells (Hematoxylin Eosin stain, original magnification ×400).

On admission, neurological examination demonstrated marked deep sensory disturbance in distal portions extremities. The MR imaging revealed a circumscribed mass lesion located on the medulla oblongata. The mass was hyperintense on T2-weighted images, isointense on T1-WI and enhanced homogeneously with gadolinium-diethylenetriamine penta-acetic acid. Examination of the cerebrospinal fluid revealed slightly elevated protein. Analysis of cells confirmed a significant lymphocytosis 86.5% (T lymphocytes: 95.7%, CD4/CD8: 5.86). Her electrocardiogram (ECG) showed complete atrioventricular (AV) block. A computed tomography (CT) of neck and chest revealed bilateral supraclavicular, hilar, and mediastinal lymphadenopathy. The patient underwent permanent pacemaker insertion immediately. Transbranchial needle aspiration was subsequently performed. Pathological examination revealed noncaseating granuloma consisting of epithelioid cells, lymphocytes, and rare multinucleated giant cells (Fig. 1). Based on these findings, pathological diagnosis was consistent with sarcoidosis.

**Figure 2:**
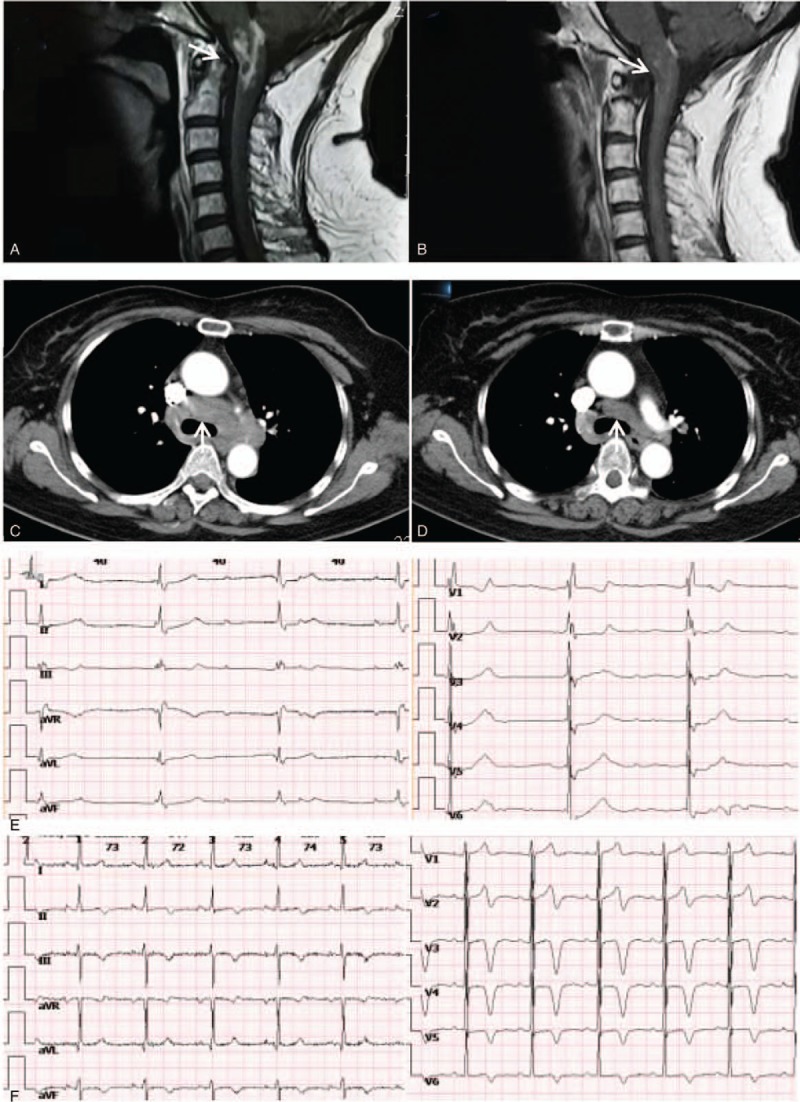
Before steroid therapy, MR imaging after gadolinium enhancement showing a mass on medulla oblongata and upper cervical cord (**A**, arrow), and CT showing lymphadenopathy (**C**, arrow). The initial ECG showed complete AV block and CRBBB (**E**). After being on steroid therapy, MR imaging after gadolinium enhancement showing nearly complete resolution of the granulomatous lesion (**B**, arrow), and CT showing the enlarged lymph nodes having become smaller (**D**, arrow). The ECG changed to normal (**F**). AV = atrioventricular, CT = computed tomography, ECG = electrocardiogram, MR = magnetic resonance.

The patient was treated with oral prednisone 60 mg/day for 4 weeks. Threedays after starting prednisone therapy, hiccough disappeared and numbness of extremities was relieved. Four weeks after starting prednisone therapy, follow up ECG and imagines showed marked improvements (Fig. 2). The dose of prednisone was tapered to 20 mg/day during 5 months, and no recurrence occurred.

## 3 Discussion

Neurosarcoidosis is primarily leptomeningeal inflammatory exudate extending from the subarachnoid space into the brain or spinal parenchyma. Locations have been reported commonly for leptomeninges, brain parenchyma, peripheral nerves, and spinal cord.^[1]^ The diagnosis of intracranial neurosarcoidosis is challenging and can be mistaken for primary brain tumors and infectious disorders of the central nervous system,^[5]^ not only because of nonspecific clinical presentations and imaging findings, but also because of high risk of obtaining specimens from the nervous system. If neurosarcoidosis is suspected, the patient should be evaluated for evidence of extraneural diseases because obtaining nerve tissue for diagnosis is often difficult. Considering biopsy specimens are sometimes unavailable, the diagnosis of isolated neurosarcoidosis is quite difficult. For the patients who were suspected to be isolated neurosarcoidosis, close follow-up was strongly recommended and empirical treatment with corticosteroids could be considered.

In present case, the medulla oblongata lesion localization and the related intractable hiccoughs is extremely rare. Hiccoughs occur due to central and peripheral causes. The central neuroanatomical localization of hiccoughs was hypothesized to lie in the medulla oblongata.^[2]^ In the absence of a clear peripheral lesion that could affect the diaphragm, the diagnosis of neurosarcoidosis involving the brainstem should be considered in patients with sarcoidosis.

Although corticosteroids are the 1st line agents for the treatment of sarcoidosis, there is no proof of survival benefit from corticosteroid treatment. Immunosuppressive agents, mostly methotrexate, are often used as a second-line agent in refractory cases and/or if there are significant steroid side effects.^[6,7]^ Resection of a central nervous system mass lesion is rarely indicated. Early recognition of this lesion may lead to appropriate treatment with steroids and avoid needlessly surgery. Complete AV block as a manifestation of cardiac sarcoidosis is regarded as a serious condition requiring prompt therapy.^[8]^ The results of some recent studies highlight the importance of early initiation of corticosteroids therapy before progression to complete AV block or advanced left ventricle dysfunction.^[8,9]^ While, in patients with advanced or complete AV block, pacemaker implantation is recommended. The studies showed that patients with cardiac and neurological involvement have a poorer outcome and a less favorable course.^[6]^ Although our patient has had a 5 moths duration recovery, we are afraid that a relapse may be inevitable.

## Author contributions

**Resources:** Zhuochao Ren.

**Writing – original draft:** Xiyuan Chen.

**Writing – review & editing:** Xiaojun Huang.

## References

[1] Lee JH Takai K Ota M Isolated neurosarcoidosis in the medulla oblongata involving the fourth ventricle: a case report. Br J Neurosurg 2013;27:393–5. 23167667 10.3109/02688697.2012.741736

[2] John S Parambil J Culver D Medullary neurosarcoidosis presenting with intractable hiccoughs. J Clin Neurosci 2012;19:1193–5. 22613488 10.1016/j.jocn.2011.11.031

[3] Caneparo D Lucetti C Nuti A A case of sarcoidosis presenting as a non-specific intramedullary lesion. Eur J Neurol 2007;14:346–9. 17355560 10.1111/j.1468-1331.2006.01527.x

[4] Mahadewa TGB Nakagawa H Watabe T Intramedullary neurosarcoidosis in the medulla oblongata: a case report. Surg Neurol 2004;61:283–7. 14985005 10.1016/S0090-3019(03)00398-7

[5] Pawate S Moses H Sriram S Presentations and outcomes of neurosarcoidosis: a study of 54 cases. Int J Med 2009;102:449. 10.1093/qjmed/hcp042 19383611

[6] Birnie DH Nery PB Ha AC Cardiac Sarcoidosis. J Am Coll Cardiol 2016;68:411–21. 27443438 10.1016/j.jacc.2016.03.605

[7] Nagai S Yokomatsu T Tanizawa K Treatment with methotrexate and low-dose corticosteroids in sarcoidosis patients with cardiac lesions. Intern Med 2014;53:2761. 25447669 10.2169/internalmedicine.53.3120

[8] Yodogawa K Seino Y Shiomura R Recovery of atrioventricular block following steroid therapy in patients with cardiac sarcoidosis. J Cardiol 2013;62:320–5. 24016620 10.1016/j.jjcc.2013.07.007

[9] David H Pablo B Andrew C Cardiac sarcoidosis. J Am College Cardiol 2016;68:411–21. 10.1016/j.jacc.2016.03.605 27443438

